# The role of innate immunity in mucopolysaccharide diseases

**DOI:** 10.1111/jnc.14632

**Published:** 2018-12-13

**Authors:** Helen Parker, Brian W. Bigger

**Affiliations:** ^1^ Stem Cell and Neurotherapies Division of Cell Matrix Biology and Regenerative Medicine Faculty of Biology, Medicine and Health University of Manchester Manchester UK

**Keywords:** heparan sulphate, inflammasome, inflammation, innate immunity, lysosomal dysfunction, mucopolysaccharidosis

## Abstract

Mucopolysaccharidoses are lysosomal storage disorders characterised by accumulation of abnormal pathological glycosaminoglycans, cellular dysfunction and widespread inflammation, resulting in progressive cognitive and motor decline. Lysosomes are important mediators of immune cell function, and therefore accumulation of glycosaminoglycans (GAGs) and other abnormal substrates could affect immune function and directly impact on disease pathogenesis. This review summarises current knowledge with regard to inflammation in mucopolysaccharidosis, with an emphasis on the brain and outlines a potential role for GAGs in induction of inflammation. We propose a model by which the accumulation of GAGs and other factors may impact on innate immune signalling with particular focus on the Toll‐like receptor 4 pathway. Innate immunity appears to have a dominating role in mucopolysaccharidosis; however, furthering understanding of innate immune signalling would have significant impact on highlighting novel anti‐inflammatory therapeutics for use in mucopolysaccharide diseases.

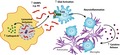

This article is part of the Special Issue “Lysosomal Storage Disorders”.

**Abbreviations used**
GAG: glycosaminoglycan
HS: heparan sulphate
IL‐1: interleukin‐1
MPS: mucopolysaccharidosis
PD: Parkinson's disease
TLR: Toll‐like receptor
TNF: tumour necrosis factor

## Mucopolysaccharidoses

Mucopolysaccharidoses (MPS) are inherited lysosomal storage diseases mainly affecting children, caused by deficiencies in lysosomal enzymes degrading glycosaminoglycans (GAG). They are part of a larger class of lysosomal storage diseases caused by deficiencies in over 50 lysosomal hydrolases and other lysosomal functions. Mutations in the enzymes that regulate lysosomal processes lead to an initial accumulation of un‐degraded substrate within the lysosomes (Vitner *et al*. 2010). Lysosomes are essential cellular components regulating cellular homeostasis, including nutrient and amino acid sensing, cell growth and cell catabolism and are sensitive to perturbation (Sancak *et al*. 2010; Zoncu *et al*. 2011). Whether directly connected or not, progressive accumulation of primary and secondary storage substrates appears to drive lysosomal, cellular and organ dysfunction that usually includes inflammatory processes (Ballabio and Gieselmann 2009). Over two‐thirds of lysosomal storage diseases (LSDs) involve CNS dysfunction, presenting as progressive cognitive and motor decline (Schultz *et al*. 2011), and the MPS diseases are no exception. This review will address the role of storage materials including glycosaminoglycans, in particular heparan sulphate (HS) in the induction of Toll‐like receptor 4 (TLR4) innate immune responses in MPS. In particular, we provide a model by which MPSIII HS can activate a TLR4 inflammatory response. Secondly, we describe the potential role of the NLRP3 inflammasome in inducing inflammation in MPS, with particular focus on lysosomal membrane permeability, cathepsin B release and disruption in ion homeostasis.

MPS diseases are all caused by a mutation in a lysosomal enzyme responsible for the degradation of GAGs (Valstar *et al*. 2008), resulting in lysosomal and extracellular accumulation of partly degraded GAG sugars. GAGs are linear sugar molecules, composed of high‐molecular weight polysaccharides with various disaccharide repeating units (Clarke 2008). GAGs usually occur in proteoglycan structures, and include HS, chondroitin sulphate (CS), dermatan sulphate (DS), keratan sulphate (KS) and hyaluronan (HA). To degrade these large molecules, individual sugars are removed from the end of the molecule by various enzymes, including lysosomal hydrolases. In MPS, GAG oligosaccharide accumulation occurs within the lysosomes of cells throughout the body resulting in numerous clinical features (Tomatsu *et al*. 2005), determined by the type of GAG accumulated (Table 1). MPS subtypes accumulating HS present with predominantly neurological‐associated symptoms, including neuroinflammation, behavioural disturbances and dementia. Those accumulating KS, CS and DS produce various non‐neuronopathic clinical features including skeletal and visceral organ pathologies, for example joint deformities, hepato‐splenomegaly and cardiac valve thickening and also include inflammation. Those accumulating HA present with soft tissue nodules surrounding joints, with short stature and mild coarse facial features as in other MPS disorders (Wraith 1995). Although the symptoms exhibited by MPS patients differ depending on the GAG accumulated (Vitner *et al*. 2010), reduced life expectancy are a commonality in all subtypes (Muenzer 2011). Several clinicians have observed that patients often show an exacerbation in disease following a cold or flu‐like infection, suggesting that inflammation may play a significant role in disease progression.

**Table 1:** Mucopolysaccharidoses are classified into several subtypes

Type	Eponym	Enzyme deficiency	GAG	Symptoms
MPSI	Hurler Hurler‐Scheie Scheie	α‐l‐iduronidase	DS/HS	Coarse facial features Short stature +/−Cognitive decline Skeletal abnormalities Hepatosplenomegaly Joint stiffness Cardio‐respiratory disease Recurrent infection
MPSII	Hunter	Iduronate‐2‐sulfatase	DS/HS	Coarse facial features Short stature +/−Cognitive decline Skeletal abnormalities Joint stiffness Hepatosplenomegaly Cardio‐respiratory disease
MPSIII	Sanfilippo A Sanfilippo B Sanfilippo C Sanfilippo D	*N*‐sulfoglucosamine sulfohydrolase α‐*N*‐acetylglucosaminidase Heparan‐α‐glucosaminide *N*‐acetyltransferase *N*‐acetylglucosamine‐6‐sulfatase	HS	Behavioural problems Cognitive decline Hearing loss
MPSIV	Morquio A Morquio B	Galactosamine‐6‐sulfatase β‐galactosidase	KS/C6‐S KS	Skeletal abnormalities
MPSVI	Maroteaux‐Lamy	*N*‐acetylgalactosamine 4‐sulfatase	DS	Coarse facial features Joint contractures Hepatosplenomegaly Cardio‐respiratory disease
MPSVII	Sly	β‐glucuronidase	DS/HS/ C4‐,6‐S	Coarse facial features Short stature Cognitive decline Skeletal abnormalities Cardio‐respiratory disease
MPSIX	Natowicz	Hyaluronidase	Hyaluronan	Short stature Frequent ear infections

MPS, mucopolysaccharidosis; DS, dermatan sulphate; HS, heparan sulphate; KS, keratan sulphate; C6‐S, chondroitin 6‐sulphate; C4‐,6‐S, chondroitin 4‐,6‐sulphate; GAS, glycosaminoglycan.

## Pathology of MPS

MPS diseases in general are characterised by increased storage of GAGs and other abnormal substrates, including but not limited to GM gangliosides, cholesterol, amyloid beta, hyperphosphorylated tau and α‐synuclein. There are probably many more that are as yet undescribed. The accumulation of these substrates has been associated with widespread inflammation, in particular CNS and joint inflammation as well as the release of various pro‐inflammatory immune mediators and autophagy dysfunction. Oxidative stress, abnormal mitochondrial function and disruption in ion homeostasis also play a role in disease pathogenesis.

## Glycosaminoglycans and their role in MPS

Glycosaminoglycans, classically termed ‘mucopolysaccharides’, are the primary storage substrate in MPS. In order to understand how GAGs, which accumulate in MPS may be linked to inflammation we need to understand the structure and signalling capabilities of GAGs.

GAG chains are diverse and are known to play a role in inflammation; however, the complexities underlying how GAGs lead to inflammation in disease settings remains poorly understood. To date, there are four families of GAG typically described; long chain sugars containing repeating disaccharide units of amino sugars and uronic acids (Taylor and Gallo 2006; Gulati and Poluri 2016). These four families include HS/heparin, CS/DS, KS and HA. HS, CS/DS and KS are arranged onto proteoglycan cores during synthesis (Li *et al*. 2012) and transported to the cell membrane, unlike HA which is directly transported into the extracellular matrix through specific transmembrane domains. HS is most notably associated with the cellular membrane of endothelial cells, and is regarded as pivotal in the control of immune cell adhesion and extravasation during inflammation and disease (Lever and Page 2001; Moon *et al*. 2005). GAGs, and in particular HS, have also been seen to influence the production of cytokines and chemokines, and play a dominant role in growth factor activation and signalling (Tanaka *et al*. 1993; Lever and Page 2001; Campo *et al*. 2009). HS has significant involvement in the regulation of chemokine and cytokine gradients produced by cells that have been stimulated by pro‐inflammatory cytokines such as interleukin‐1 (IL‐1) and tumour necrosis factor‐α (TNF‐α) (Tanaka *et al*. 1993; Celie *et al*. 2009).

During inflammatory disease or injury, GAGs may be released from their proteoglycan cores either by direct or enzymatic heparanase cleavage (Reiss *et al*. 1986); these small GAG fragments have been found to be highly immunogenic in diseases such as cancer, arthritis, asthma, other autoimmune diseases, neurodegenerative disease and also MPS (Salvatore *et al*. 2000; Lever and Page 2001; Wang and Roehrl 2002; Coutinho *et al*. 2012). Recently, GAG fragments have been highlighted as a potential endogenous danger‐associated molecular pattern (DAMPs); similar to bacterial infections, it may initiate an innate immune signalling event to which the host responds. Heparanases are endoglycosidases which catalyse the specific breakdown of HS extracellularly. In doing so, they reduce HS chain length and affect HS signalling. The levels of heparanase are significantly increased during inflammatory disease, in particular cancer metastasis and neuroinflammation (O'Callaghan *et al*. 2018), and are able to cleave low‐molecular weight HS fragments which are heavily sulphated (Escobar Galvis *et al*. 2007). The binding of many growth factors, chemokines and other immune regulators depend on the highly sulphated ‘heparin‐like’ domain of HS.

The accumulation of HS is one of the primary pathological features of MPSI, MPSII, MPSIII and MPSVII, a direct result of lysosomal hydrolase deficiencies. HS levels are typically increased in MPS patient's plasma and CSF (Tomatsu *et al*. 2005; Naimy *et al*. 2016); this HS storage is mirrored well by the MPS animal models. HS storage is evident in the CNS of MPS murine models (Wilkinson *et al*. 2012; Martins *et al*. 2015; Roca *et al*. 2017; Tanaka *et al*. 2018). Many have observed an abundant accumulation of HS in particular areas of the CNS including the cerebral cortex, dentate gyrus, cerebellum and brainstem (Arfi *et al*. 2011; Wilkinson *et al*. 2012); however, the deposition of HS is widespread. There is an accumulation of partially degraded HS in the lysosomal compartments of neurons and microglia (McGlynn *et al*. 2004; Ohmi *et al*. 2011; Martins *et al*. 2015) in MPSIII models, although increased levels of HS are also associated with the extracellular matrix in MPSI (Watson *et al*. 2014). The accumulation of HS is not confined to the CNS; accumulation of HS is apparent throughout the periphery in MPSI, MPSII, MPSIII and MPSVII (Cardone *et al*. 2006; Holley *et al*. 2011, 2018; Roca *et al*. 2017).

The absence of HS degrading lysosomal enzymes also results in an increase in the amount of MPSIII HS with characteristic non‐reducing end structures (Lawrence *et al*. 2012). It is unlikely that these non‐reducing ends play a role in MPSIII disease progression, but analysis of the non‐reducing ends does provide a useful biomarker to monitor the efficacy of therapeutic treatments based on enzyme replacement therapy.

The enzymatic events during GAG synthesis strongly affect the how GAGs bind to their specific co‐factors and dictate their role in homeostasis and disease (Sarrazin *et al*. 2011). During polymerisation, GAG chains undergo several modifications, including sulphation of amino sugars and uronic acids, these enzyme modifications play an important role in directing GAG variability and activity (Kreuger and Kjellen 2012). MPSI, MPSII, MPSIIIA, MPSIIIB and MPSIIIC mice all exhibit a significant increase in the level of sulphation of HS disaccharides (Wilkinson *et al*. 2012; Gleitz *et al*. 2018; Holley *et al*. 2018; Tordo *et al*. 2018). Many HS‐binding co‐factors exhibit a binding preference for regions containing 2*‐O‐*sulphation. Significant increases in HexA(2S)‐GlcNS(6S) and HexA(2S)‐GlcNS HS disaccharides are evident in MPSIIIA and MPSIIIB mouse brains (Wilkinson *et al*. 2012; Sergijenko *et al*. 2013; Holley *et al*. 2018). 2‐*O*‐sulphation of HS has already shown to mediate C‐X‐C motif chemokine 12 (Stromal Derived Factor 1)‐mediated Hematopoietic stem cell migration in MPSI (Watson *et al*. 2014); however, highly sulphated HS may indeed play a role in mediating a variety of signalling and inflammatory pathways in other MPS subtypes.

DS in a similar way to HS acts to modify various biological responses. The complexity of DS varies in a similar fashion to HS; the length of the DS chain, IdoA content and placement, the levels of sulphation and the variety of core proteoglycans to which it attached all dictate it's binding and signalling ability. DS accumulation is most typically associated with somatic and skeletal involvement in MPSI, MPSII, MPSVI and MPSVII. Whilst DS storage is not a primary feature of MPSIII, and its storage is better associated with MPSI and MPSII, increases in DS are evident in MPSIII patient samples and MPSIII animal models. Chondroitin/DS levels in MPSIII fibroblasts were elevated 2–5‐fold above normal dermal fibroblasts (Lamanna *et al*. 2011); this accumulation was suggested to be a result of IDS (DS degrading enzyme) activity inhibition by MPSIII HS. Regardless of the mechanism, similar increases in DS have been observed in the newborn MPSIII dried blood spots (de Ruijter *et al*. 2012) and in the liver from MPSIIIB mice (Holley *et al*. 2018). The role of GAGs in MPS pathology will be discussed within this review, and we will highlight a model which demonstrates how highly sulphated HS in particular may elicit an immune response in MPSIIIA.

## GM gangliosides in MPS

GM gangliosides are primary substrates in several lysosomal diseases with a significant inflammatory component such as GM1 and GM2 gangliosidosis (Jeyakumar *et al*. 2003; Kasperzyk *et al*. 2005; Baek *et al*. 2008). The loss of lysosomal enzyme activity as observed in MPS is also able to modify the activity of other lysosomal hydrolases (Clarke 2008). Li and associates demonstrated a decrease in sialidase activity in MPS, which is likely a contributing factor to an increase in glycosphingolipid storage observed throughout MPS brains (Li *et al*. 1999; Walkley 2004; Walkley and Vanier 2009). Accumulation of GM2 and GM3 gangliosides has been demonstrated in MPS III animal models. GM2 and GM3 ganglioside accumulation has been co‐localised to neurons and microglia and is particularly evident in cortical layers II, III, V, the amygdala and the hippocampus (McGlynn *et al*. 2004; Crawley *et al*. 2006; Ausseil *et al*. 2008; Ohmi *et al*. 2011; Wilkinson *et al*. 2012; Martins *et al*. 2015). McGlynn and colleagues showed accumulation of GM2 and GM3 in separate vesicles within affected neurons. Their suggestion was that other mechanisms including trafficking and/or altered synthesis of secondary stored gangliosides could be important factors in MPS (McGlynn *et al*. 2004). However, it is unclear why GM2 and GM3 gangliosides are present when other GM gangliosides are not (Walkley 2004). GM2 and GM3 gangliosides have been postulated to alter axon morphology and synaptic transmission (Clarke 2008), as well as initiate various inflammatory processes which may be a secondary factor in MPS disease pathogenesis.

## Cholesterol in MPS

Total levels of cholesterol do not appear to be increased in the brains of MPSIII animal models; however, labelling of the free unesterified form of cholesterol using Filipin III histology demonstrates significant accumulation. This accumulation is confined to the lysosomes of neurons and microglia (McGlynn *et al*. 2004), but has also been well documented in a variety of lysosomal storage diseases including other MPS subtypes, GM gangliosidoses and Wolman's disease (Walkley 2004). Again, all of these diseases share commonality in inflammatory responses, although the precise mechanisms may vary.

## Other abnormal proteins in MPS

Increased levels of protein markers, including those associated with Alzheimer's disease and other tauopathies have been observed in neurons of specific brain areas of MPSIIIA and MPSIIIB mice involved in learning and memory, including dentate gyrus and medial entorhinal cortex. Deposition of lysozyme, hyperphosphorylated tau, phosphorylated tau kinase, GSK3B, and amyloid‐β and amyloid precursor protein are all evident in the brains of MPSIII mice (Ohmi *et al*. 2009, 2011; Beard *et al*. 2017). MPSIIIC mouse brains also show elevations in these markers although there is typically lower accumulation than similarly aged MPSIIIA and MPSIIIB mice (Ohmi *et al*. 2011; Beard *et al*. 2017). MPSIII patient brains demonstrate cytoplasmic amyloid‐β_(1–40)_ staining throughout the brains, and 3‐fold increase in soluble amyloid‐β_(1–40)_ (Ginsberg *et al*. 1999).

Alpha‐synuclein aggregation, typically a primary characteristic of Parkinson's disease (PD) is also a secondary feature of MPSIII. Immunostaining of MPSIIIA and MPSIIIB patient brain sections showed α‐synuclein accumulation with neurons localised within the cortex, hippocampus, thalamus and substantia nigra (Hamano *et al*. 2008; Winder‐Rhodes *et al*. 2012). Similar aggregation of α‐synuclein was evident in MPSIIIA mice as early as 3 weeks of age (Beard *et al*. 2017). One study suggests a possible association between a mutation in NAGLU (affected enzyme in MPSIIIB) and the susceptibility to PD. It remains unknown whether mutations in other lysosomal hydrolases are associated with risk for PD.

## Neuroinflammation in MPS

The extent of neuroinflammatory and neurodegenerative changes in MPS has been observed in murine models of MPS. The MPSIII mouse models recapitulate aspects of patient disease, demonstrating a similar phenotype as outlined above, as well as pathological and neuroimmunological similarities (Wilkinson *et al*. 2012).

MPSIII diseases are usually referred to as ‘*neurodegenerative’*. There is a small body of evidence that suggests age‐related progressive loss of neurons in MPSIIIB and MPSIIIC mice (Heldermon *et al*. 2007; Martins *et al*. 2015); however, neuronal loss is not consistently observed in MPSIII animal models. Neuronal loss and cerebral atrophy are detected in brains of MPSIII patients with magnetic resonance imaging and upon autopsy (Valstar *et al*. 2008; Sharkia *et al*. 2014), demonstrating the phenotype variation in patients versus murine models. However, the functional role of neuronal cell death in MPSIII clinical disease onset and progression is neither well established, nor understood. Several factors probably combine to cause clinical manifestation of MPSIII disease and there is no doubt that neuronal dysfunction is part of this, but it could be any one of several factors including defective synaptic transmission, abnormal axonal features, chronic neuroinflammation and defective signal transduction.

Both microglial and astrocyte activation are present throughout the brain of MPSI, MPSII and MPSIII mouse models, as demonstrated by increases by isolectin‐B4 and Glial Fibrillary Acidic Protein (GFAP) immune‐positive staining (Villani *et al*. 2007; Arfi *et al*. 2011; Wilkinson *et al*. 2012; Martins *et al*. 2015; Roca *et al*. 2017; Gleitz *et al*. 2018; Tordo *et al*. 2018). Li and associates also observed an increase in GFAP mRNA in the cerebral cortex, hippocampus and cerebellum of MPSIIIB mice (Li *et al*. 2002). Other studies have demonstrated an increase in microglial activation via the up‐regulation of Iba1 and CD11b; however these are poor markers in selecting what state of activation microglia reside in, apart from indicating an activated amoeboid morphology (Holtman *et al*. 2017).

Pro‐inflammatory cytokines such as IL‐1, TNF‐α, MCP‐1 and MIP‐1α are all shown to be elevated in the CNS of the MPSIIIA murine model (Ausseil *et al*. 2008; Arfi *et al*. 2011; Wilkinson *et al*. 2012; Martins *et al*. 2015) similar to the cytokine profiles observed in murine models of Gaucher disease and Krabbe (Vitner *et al*. 2016). Most likely the majority of these cytokines are produced by activated‐microglia, but activated‐astrocytes and even neurons are also capable of propagating pro‐inflammatory cytokine signalling. Significant increases in MCP‐1, MIP‐1α and IL‐1α protein levels have been observed in MPSI and MPSIII mouse brains (Wilkinson *et al*. 2012; Guo *et al*. 2018; Holley *et al*. 2018). Arfi and associates, and Ausseil and associates showed an increase in MIP‐1α transcript levels, as an early marker of disease progression in mouse models of MPSIIIA and MPSIIIB (Ausseil *et al*. 2008; Arfi *et al*. 2011). TNF‐α and TNFR1 gene expressions were up‐regulated in brains of MPSIIIB mice by 3 months of age when compared to wild‐type (Arfi *et al*. 2011; Trudel *et al*. 2015). Increases in serum TNF‐α are evident in MPSVII mice (Simonaro *et al*. 2010), as well as increases in synovial fluid TNF‐a from MPSVII dogs (Simonaro *et al*. 2008). These changes in gene and protein expression suggest the activation of signalling cascades downstream from innate immune TLRs and IL‐1 receptors, which would both contribute to TNF‐α production. IL‐1β transcript levels were highlighted as a later marker of neuroinflammation in MPSIIIA and MPSIIIB. IL‐1β transcript levels were up‐regulated at 2–4 months of age, and carried on increasing with age in both MPSIIIA and MPSIIIB brains (Ausseil *et al*. 2008; Arfi *et al*. 2011). IL‐1β protein expression was also increased in synovial fluid from MPSVII dogs, and MPSVI rat fibroblast‐like synoviocytes (Simonaro *et al*. 2001, 2008, 2010). Understanding the pathogenic mechanisms leading to cytokine secretion and the contribution of pro‐inflammatory mediators will be critical in highlighting future anti‐inflammatory therapies for MPS.

## The role of the periphery in inflammation

Whilst the focus of this review is neuroinflammation, it would be unwise to discount the role of the periphery in influencing CNS events – particularly where cytokine levels in the periphery often vastly exceed those centrally in the brain, and cytokines are able to move freely across the blood brain barrier. The data from MPSVI rats and MPSVII mice (Simonaro *et al*. 2001, 2008, 2010) suggest that peripheral inflammation in MPS disease may largely be driven and controlled by TNF‐α. This may not be the case in the brain where TNF‐α can be subordinate to other cytokine responses such as IL‐1. Nonetheless, there is no doubt that peripheral inflammation can influence central events, and thus targeted strategies should address both central and peripheral inflammation together.

## TLR4 inflammatory signalling pathways in MPS

**Figure 1:**
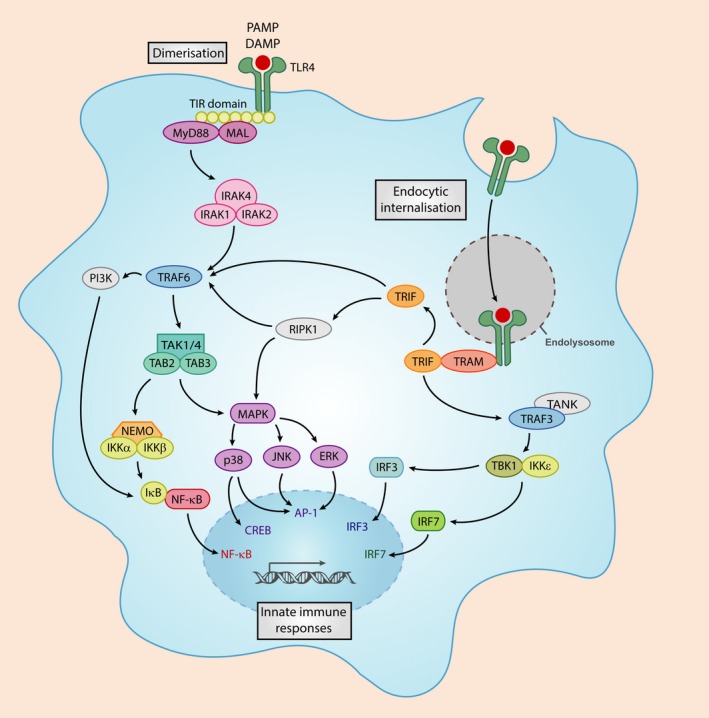
Toll‐like receptor 4 (TLR4) signalling in response to a danger signal. PAMPs and danger‐associated molecular patterns (DAMPs) bind TLR4 resulting in MyD88 adaptor protein recruitment. This leads to the assembly of an IRAK4/IRAK1/IRAK2/TRAF6 complex. The IRAK1/TRAF6 complex binds a secondary complex, TAB2/TAK1/TAB1. This allows TRAF6 to dislocate from IRAK1 and move into the cytoplasm of the cell, whereby it can lead to the activation of various transcription factors, and initiation of a pro‐inflammatory response. Endocytosed‐TLR4 bound to its respective ligand is able to signal via TRIF in order to transcribe interferon transcription factors.

HS chains and proteoglycans are capable of promoting an inflammatory response, including the production of TNF‐α and IL‐1β (Kodaira *et al*. 2000). Soluble HS fragments can be detected via TLR4 in order to monitor tissue well‐being (Johnson *et al*. 2002). However, proteoglycan‐bound HS within the extracellular matrix plays a limiting role with regard to TLR4 activation, thereby normal HS may mediate TLR4 inhibition (Brunn *et al*. 2005). Once HS is released from the extracellular matrix, soluble HS fragments can assume a TLR4 agonist role and promote an immune response (Goodall *et al*. 2014). There are many aspects of HS interactions with TLR4 and its counterparts that remain unclear; however, it is clear that HS can play an important role in facilitating innate immune responses. If HS acts as a DAMP, similar to that of bacterial pathogens, it may elicit its immune response via the pathway illustrated in Fig. 1.

A causative factor in MPS neuroinflammation is likely the accumulation of HS in the CNS, likely through its interactions with TLRs and other immune regulators; many of which have been revealed to comprise HS‐binding motifs, such as TLR4, TLR2, Interleukin 1 Receptor Associated Kinase 1 (IRAK1) and the inflammasome receptor NLRP3 (Simon Davis and Parish 2013).

The ability of HS to regulate co‐factor binding and signalling significantly relies on the degree of sulphation and sulphation patterning. The composition of heparan sulphate was analysed in a neuropathological study by Wilkinson and associates utilising MPSI, MPSIIIA and MPSIIIB mice. They observed an up‐regulation in the sulphation of various HS disaccharides, with significant increases in HexA(2S)‐GlcNS(6S) and HexA(2S)‐GlcNS in MPS when compared to wild‐type (Wilkinson *et al*. 2012). A similar HS sulphation pattern was observed in a mouse model of MPSII (Gleitz *et al*. 2018). These HS disaccharides may contribute to the initiation of various signalling pathways in MPS; especially if these highly sulphated disaccharides have the potential to act as endogenous DAMPs (Archer *et al*. 2014) both intracellularly and extracellularly.

**Figure 2:**
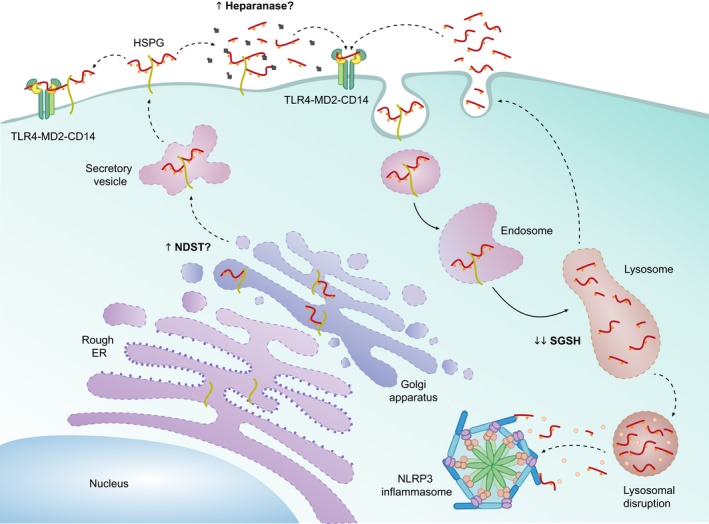
Proposed role of MPSIIIA heparan sulphate (HS) in innate immunity. HS is synthesised in the endoplasmic reticulum‐Golgi, where it undergoes chain modification events. It is likely that MPSIIIA HS is highly sulphated due to an increase in activity of the chain modification enzyme NDST. HS bound to a proteoglycan (HSPG) is exocytosed to the cell membrane. In its HSPG form it may interact with the TLR4‐MD2‐CD14 complex to propagate an inflammatory response. HS may also be cleaved by heparinase, an endoglycosidase which has been seen to be up‐regulated in cancer metastasis and neuroinflammation. Increased heparanase activity would digest HS to short fragments which may have the potential to bind the TLR4‐MD2‐CD14 complex. In order to degrade HS, HSPGs are endocytosed and digested within the endolysosome, the deficiency in SGSH which is the primary cause of MPSIIIA, would result in HS fragments which are highly sulphated with a sulphated non‐reducing end residue. These fragments may either undergo exocytosis and subsequent binding to the TLR4 complex, or be released intracellularly due to lysosomal destabilisation and directly activate the NLRP3 inflammasome.

Taking into account that there is an accumulation of highly sulphated HS in MPSIII and other MPS subtypes, we postulate that the sulphation patterning of MPS HS tightly regulates innate immune responses (Fig. 2). We already know that the sulphation of HS is carefully regulated during HS synthesis in the ER–Golgi pathway. The modification of the HS backbone starts with *N*‐deacetylation/*N*‐sulphation of GlcNac residues by the N‐Deacetylase/N‐Sulfotransferase (NDST) enzyme in clusters along the chain. NDSTs clearly play a key role in the formation of ligand‐binding domains because many of the subsequent modifications then occur at the sites of *N*‐sulphation, leading to sulphated ‘patches’ along the chain (Esko and Lindahl 2001). The *N*‐sulphated regions can then undergo epimerisation and 2‐*O*‐sulphation, thus the pattern of *N*‐sulphation dictates the position of the highly modified *S*‐domains, and affect positioning of binding sites. Interestingly, increased activity in the HS modification NDST enzyme was observed in MPSI murine brain (Holley *et al*. 2011). Whether similar changes to the activities of MPSIII HS‐synthesis enzymes are present, remain unclear; however, targeting NDST activity could reduce HS sulphation and alleviate inflammation associated with MPS. Others have postulated that the non‐reducing end structure of HS, the uncleaved linkage present in excess due to the absence of the lysosomal enzyme, which is unique in each MPS disease, would be more likely to stimulate inflammation and better explain the differences in behaviour observed in different MPS subtypes (Lawrence *et al*. 2012). Severe MPSI patients are vastly different clinically to MPSII patients for example despite both accumulating HS and DS, although MPSIII subtypes are all clinically very similar.

Both Simonaro and associates, and Ausseil and associates hypothesised that lysosomal and/or extracellular GAG storage in the MPS disorders can induce inflammation by activating TLR4 signalling pathway. Simonaro and colleagues postulated that GAGs induce pathological changes in MPS via activation of the lipopolysaccharide‐mediated TLR4 signalling pathway, which required MyD88, LBP and CD14. Indeed they demonstrated increased protein expression of TLR4, MyD88 and LBP in rat fibroblast‐like synoviocytes and chondrocytes as well as transcriptional up‐regulation of CD14 in MPSVI fibroblast‐like synoviocytes (Simonaro *et al*. 2008). They also put forward the hypothesis that activation of the TLR4 pathway can also be induced by GAG fragments, in particular HA interacting with CD44, a HA‐binding cell surface glycoprotein receptor and MyD88, providing support for the concept that this pathway is activated by GAG storage in the MPS disorders (Simonaro *et al*. 2010).

Further to this Ausseil and associates demonstrated that pathogenic MPSIIIB patient HS could elicit a pro‐inflammatory response in mouse microglial cells *in vitro*, with significant increases in TNF‐a, IL‐1β and MIP‐1a expression. In contrast, GAGs extracted from normal urine had no pro‐inflammatory effect. These MPSIIIB GAG‐dependent immune responses were dramatically reduced in TLR4 and MyD88 knockout cells. Furthermore, MPSIIIB mice were bred with TLR4^−/−^ or MyD88^−/−^ knockout mice to identify whether the progression of neuroinflammation was dependent on either of these proteins. Lack of microglial priming in TLR4 and MyD88‐deficient MPSIIIB mice suggests early microglial activation is dependent on a TLR4/MyD88 signalling pathway, and is likely driven by HS alone. As the disease progresses and accumulation of secondary storage substrates become apparent, microgliosis is evident in both TLR4 and MyD88‐deficient MPSIIIB mice, suggesting a role for other drivers of microglial activation, including GM gangliosides. Interestingly at 3 months of age *IL1b* transcriptional expression is up‐regulated in MPSIIIB mice; however, knockout or TLR4 or MyD88 abolishes *IL1b* expression, suggesting that early in disease progression *IL1b* priming is regulated by TLR4; however, as the disease progresses, TLR4/MyD88 deficiency has no effect on *IL1b* expression in MPSIIIB (Ausseil *et al*. 2008). This indicates that *IL1b* is being primed in response to a TLR4/MyD88‐independent pathway. This series of studies suggests that pathogenic MPSIII HS can act as a DAMP to activate a TLR4/MyD88‐dependent pathway in order to prime microglia early in disease pathogenesis (Fig. 2). The exact form of HS required to activate an immune response is unclear (Fig. 2). Firstly, highly sulphated HS bound to its proteoglycan core may interact with TLR4 at the cell surface similar to the model postulated by Li and associates (O'Callaghan *et al*. 2018). Secondly, extracellular heparanase activity may be up‐regulated as observed in cancer and other neuroinflammatory diseases, which will cleave HS from its proteoglycan core and produce inflammation inducing HS fragments (Mason *et al*. 2014). Lastly, the lysosomal enzyme defects associated with MPS result in an intralysosomal accumulation of partially degraded HS; this HS has the potential to be exocytosed and elicit immune responses via TLR4. It must be noted that HS accumulation alongside the accumulation of other secondary storage substrates and autophagic dysfunction in progressed MPSIII may activate various other neuroinflammatory pathways independent of TLR4.

## A potential role for the inflammasome in MPS pathogenesis

Lysosomes play an important role in regulating the immune response from controlling autophagy, lipid metabolism, glycosaminoglycan degradation and regulation of inflammasome‐dependent release of cytokines. The relationship between autophagy and inflammation has been recently linked to the release of IL‐1β by the inflammasome (Abdelaziz *et al*. 2015). Autophagy is a negative regulator with respect to inflammasome activation and secretion of IL‐1β, and removal of pro‐autophagy machinery from mice causes increased inflammasome‐dependent IL‐1β release (Saitoh *et al*. 2008).

**Figure 3:**
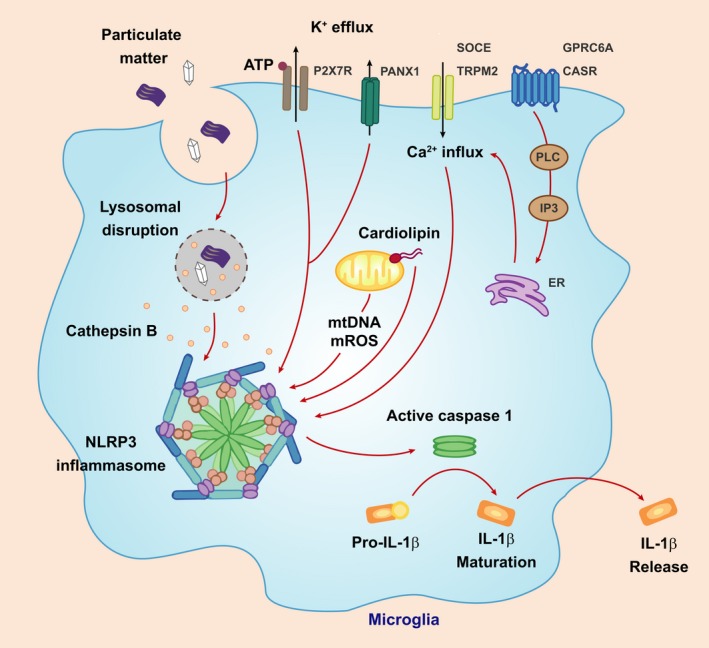
Pathways regulating NLRP3 inflammasome activation. Various stimuli, such as excessive ATP, viral RNA and particulate matter (e.g. protein aggregates or crystalloid structures) have the ability to activate the NLRP3 inflammasome and induce the maturation and secretion of interleukin‐1β (IL‐1β). An excess of ATP, and most other NLRP3 activators bring about the efflux of K^+^. An increase in intracellular Ca^2+^ from intracellular stores or via transmembrane transporters has been associated with NLRP3 inflammasome activation. The production of reactive oxygen species (mtROS), as a result of mitochondrial dysfunction is another potential driver of inflammasome activation. Lastly, particulate matter taken up via phagocytosis can lead to lysosomal membrane permeabilisation, the leakage of cathepsin B and other cysteine proteases and NLRP3 inflammasome activation.

The activation of the inflammasome is mediated by the innate immune system in response to cellular stress signals such as lysosomal dysfunction, ion homeostasis dysregulation and oxidative stress (Latz *et al*. 2013). Several families of pattern recognition receptors are important components in the inflammasome complex including Non Obese Diabetic‐like receptors. Upon sensing certain stimuli, the relevant inflammasome receptors can oligomerise into a caspase‐1 activating complex. Cleavage of pro‐caspase 1 into its mature form, results in the maturation of IL‐1β and its release into the extracellular space, whereby it can elicit pro‐inflammatory effects (Martinon *et al*. 2009; Schroder *et al*. 2010; Song *et al*. 2017). The NLRP3 inflammasome is activated in response to the widest array of stimuli. Over the last 20 years, various NLRP3 agonists that induce NLRP3 inflammasome activation have been highlighted, including ATP, crystalline and fibrillar substances, nucleic acids, fungal, bacterial or viral pathogens and to a lesser degree glycosaminoglycans (Guo *et al*. 2015). Various pathological conditions may promote the formation of these stimuli, e.g. Alzheimer's disease, Parkinson's disease, multiple sclerosis, atherosclerosis and diabetes type 2 (Song *et al*. 2017). The mechanism of NLRP3 activation supported by mouse studies includes potassium efflux, the generation of mitochondrial reactive oxygen species, the translocation of NLRP3 to the mitochondria, the release of mitochondrial cardiolipin and DNA, increased intracellular calcium, and the release of cathepsins into the cytosol after lysosomal destabilisation. The pathways known to regulate NLRP3 inflammasome activation and IL‐1β secretion are illustrated in Fig. 3.

Lysosomal membrane destabilisation is a well‐known activator of the NLRP3 inflammasome; however, the role of lysosomal membrane destabilisation in MPS has not been investigated. Recent evidence does support the role for the NLRP3 inflammasome in Gaucher disease (GD). Aflaki and associates demonstrated that impaired autophagy in GD macrophages induced sustained NLRP3 inflammasome activation and IL‐1β secretion. Indeed further suppression of autophagy in GD macrophages with 3‐methyladenine or bafilomycin A1 exacerbated the cleavage and secretion of IL‐1β, suggesting that the inflammasome could be a key component to the immune response in LSDs (Aflaki *et al*. 2016).

Cathepsin B is able to activate inflammasome complex assembly directly. Inflammasome activation has never been implicated in MPS; however, recent evidence implicates cathepsin B in the pathogenesis of MPS type I. Similar to MPSIIIA mice, cathepsin B has been shown to be over‐expressed in various tissues from MPS I mice. Gonzalez and associates found that treatment of MPS I mice with a cathepsin B inhibitor reduced aortic dilatation and heart valve thickening, and led to improvements in cardiac function. They also found that cathepsin B leaks from the lysosome in MPS I human fibroblasts (Gonzalez *et al*. 2018). With regard to the involvement of ion homeostasis in inflammasome activation, Pereira and colleagues showed that splenic lymphocytes harvested from MPS I mice released more Ca^2+^ than control mice. A lower content of intralysosomal H+ was also evidenced, suggesting an alteration of pH homeostasis.

This evidence implies that lysosomal membrane permeability, cathepsin B and altered ion homeostasis play a role in MPS I disease pathogenesis, through an unknown mechanism that is likely shared by other MPS subtypes. Indeed cathepsin B gene expression is up‐regulated in brain of MPSIIIA mice when compared to WT mice (Arfi *et al*. 2011). Taking into consideration that IL‐1 expression is up‐regulated in MPSIII, cathepsin B, a major regulator of the NLRP3 inflammasome is also up‐regulated, and that various secondary storage substrates including cholesterol and amyloid beta activate the inflammasome, it is possible that an inflammasome‐dependent pathway may be involved in MPSIII disease progression and inflammation.

## Anti‐inflammatory treatments in MPS

Many of the current treatments for MPS have been unable to reduce neuroinflammation. Treatments are therefore being developed to target neuroinflammation in MPS. Many genes associated with neuroinflammation are up‐regulated in MPS between 2 and 4 months of age, when the phenotype becomes apparent in the mouse model. Arfi and associates administered intraperitoneal high‐dose aspirin (200 mg/kg) to MPSIIIB mice for 2, 4 and 6 months three times a week. At 6 months, transcript levels for MIP‐1α, IL‐1β, GFAP and oxidative stress were significantly reduced (Arfi *et al*. 2011). However, the high‐dose of aspirin used in this study is not clinically relevant to human MPS patients. It is also unknown whether there was any reversal in MPSIIIB behaviour, as no behavioural assessment took place.

Substrate reduction therapy has been highlighted as a key treatment to reduce the levels of GAGs and inflammation in MPS. Genistein, an isoflavone found in soy products has the ability to significantly reduce HS accumulation and lysosomal compartment size in MPSIIIB mice administered 160 mg/kg/day for 9 months. The treatment also resulted in a significant reduction in staining for activated‐astrocytes and microglia in the cerebral cortex of treated MPSIIIB mice when compared to wild‐type (Malinowska *et al*. 2010). However, long‐term memory and learning tests were not performed, therefore it is difficult to assess whether genistein completely reverses cognitive decline in MPSIIIB. This treatment has since moved into clinical a phase III clinical trial.

Prednisolone, a corticosteroid has also been tested in the treatment of neuroinflammation in MPSIIIB. Three to 5 weeks old MPSIIIB mice were administered with 0.75 mg/kg prednisolone daily for 6 months, and the levels of astrocyte activation measured. A significant decrease in GFAP staining was apparent in MPSIIIB mice when compared to untreated mice. The study also assessed behavioural outcomes; treated‐mice performed significantly better in the Morris water maze test, this suggests that long‐term memory and learning were improved (DiRosario *et al*. 2009). A later study used prednisolone doses of 1 mg/kg/day from 6 weeks to 8 months of age in MPSIIIB mice and observed correction of hyperactive behaviour, and reduction of liver cytokines and IB4 Kuppfer cell activation in the liver, in the absence of any brain reduction in cytokines, astrocyte or microglial activation (Holley *et al*. 2018). This suggests that reducing peripheral inflammation may be sufficient in itself to affect central behaviour in MPS mice at least, but it is not clear if this treatment is targeted enough to have application in patients, especially given the side effects of long‐term steroid use.

TLR activation promotes the production and release of TNF‐α; pentosan polysulfate (PPS) is a TNF‐α antagonist and HS mimic, and has recently been administered to MPSVI rats models in order to reverse skeletal abnormalities. TNF‐α and MIP‐1α levels were significantly reduced in chondrocytes after 8 months treatment with PPS (Schuchman *et al*. 2013). PPS may offer the potential to reduce the TNF‐α component in MPSIIIA neuroinflammation, if it possesses blood brain permeable properties, or if injected directly into the CNS. Alternatively, it may be able to exert an effect just through reducing peripheral cytokine levels (Guo *et al*. 2018).

We propose that targeting IL‐1 or inflammasome‐dependent pathways in MPSIII as well as other LSDs over‐expressing IL‐1 could pose great benefit in the clinical setting. It must be noted that anti‐inflammatory drugs are not going to be curative but may improve quality of life and slow disease progression. They could also be used as adjuncts to enzyme replacement therapies, in order to achieve additive or synergistic benefit.

## Conclusion

We propose that accumulation of abnormal pathological substrate, alongside autophagy dysfunction and lysosomal membrane destabilisation may initiate a self‐propagating innate immune response in MPS. However, we require further understanding of the signalling pathways involved in inflammation, which in turn will highlight potential anti‐inflammatory therapeutics for use in MPS disease treatment.

## Acknowledgments and conflict of interest disclosure

BB has shares and licensed programmes in enzyme replacement stem cell gene therapy for MPSIIIA and MPSIIIB to Orchard Therapeutics Ltd. BB has shares and licensed programmes in enzyme replacement gene therapy for MPSIIIC to Phoenix Nest Inc. Neither interest competes with the content of this paper, which deals with pathophysiology and the involvement of inflammation in MPSIIIA and other LSDs. HP was supported by a PhD studentship awarded by the NRI, University of Manchester.
